# 565 Complications and Outcomes for Electrical Burn Admissions

**DOI:** 10.1093/jbcr/iraf019.194

**Published:** 2025-04-01

**Authors:** Arman Fijany, Tyler Murphy, Punit Vyas, Emily Swafford, Jordan Garcia, Robel Beyene, Stephen Gondek, Anne Wagner, Elizabeth Slater

**Affiliations:** Vanderbilt University Medical Center; Vanderbilt Burn Center; Vanderbilt University Medical Center; Vanderbilt Burn Center; Vanderbilt University Medical Center; Vanderbilt University Medical Center; Vanderbilt University Medical Center; Vanderbilt University Medical Center; Vanderbilt University Medical Center

## Abstract

**Introduction:**

Electrical burns are among the most devastating burns treated in an inpatient burn unit. While electrical injuries have been described in the literature as being a risk factor for mortality and significant complications such as deep venous thrombosis (DVT), these associations have not been well established with multi-institutional clinical data.

**Methods:**

The American Burn Association (ABA) Noncommercial Burn Research Dataset was queried for patients admitted with electrical burns from 2012-2021. Primary outcomes included death, ICU admission, compartment syndrome (abdominal and extremity), respiratory failure, pneumonia, DVT, and hospital length of stay (LOS). A chi-square analysis was performed to analyze demographic associations. A multivariate regression analysis was performed while controlling for the following covariates (age, sex, TBSA%, and inhalation injury). Data analysis was done with PyCharm 3.1 software using the pandas and Scipy.stats modules.

**Results:**

Our study included 285,544 patient encounters, 7,543 (2.6%) of which were admissions for electrical burns. Patients with electrical burns were significantly more likely to be male (p < 0.0001), white (p < 0.0001), and less likely to be homeless (p < 0.0001). When controlling for confounding variables, patients with electrical injuries had a significantly higher rate of mortality (p < 0.0001), ICU admission (p < 0.0001), compartment syndrome (p < 0.0001), DVT (p < 0.05), and hospital LOS (0.68 days, p < 0.0001).

**Conclusions:**

This is the most extensive retrospective analysis of burn data for this injury type in the literature. After controlling for confounding variables such as age, sex, TBSA%, and inhalation injuries, electrical burns remain significantly associated with more extended hospital LOS and higher rates of mortality, DVT, ICU admission, and compartment syndrome when compared to other burn injury types.

**Applicability of Research to Practice:**

Our study is the first to demonstrate a strong association between electrical burns and complications, such as DVT and compartment syndrome, that is supported by clinical data. These results can help guide clinicians in creating specific treatment protocols for those admitted with electrical burns, such as increasing DVT prophylaxis dosage, identifying compartment syndrome earlier, and prioritizing higher levels of intensive care for this high-risk population.

**Funding for the Study:**

N/A

